# Revisiting myocardial necrosis biomarkers: assessment of the effect of conditioning therapies on infarct size by kinetic modelling

**DOI:** 10.1038/s41598-017-11352-4

**Published:** 2017-09-06

**Authors:** David Ternant, Fabrice Ivanes, Fabrice Prunier, Nathan Mewton, Theodora Bejan-Angoulvant, Gilles Paintaud, Michel Ovize, Denis Angoulvant

**Affiliations:** 1 Université François Rabelais de Tours, CNRS, UMR 7292 GICC Tours, France; 2 CHRU de Tours, Laboratory of Pharmacology-Toxicology, Tours, France; 3 Université François Rabelais de Tours, EA 4245 CDG & FHU SUPORT, Tours, France; 4 CHRU de Tours, Department of Cardiology & FACT, Tours, France; 5 Université d’Angers, EA 3860 CRT, Angers, France; 6 Université Claude Bernard Lyon 1, INSERM U1060 CarMeN Lyon, France; 7 CHRU de Tours, Department of Clinical Pharmacology, Tours, France

## Abstract

Infarct size is a major predictor of subsequent cardiovascular events following ST-segment elevation myocardial infarction (STEMI) and is frequently used in clinical trials focused on cardioprotection. Approximately assessed through serial blood sampling, it can be accurately measured by imaging techniques, e.g. cardiac magnetic resonance imaging, which is the actual gold standard for infarct size determination but with limited availability in daily practice. We developed a mathematical biomarker kinetic model based on pharmacokinetic compartment models to easily and accurately estimate infarct size using individual data from five clinical trials evaluating the impact of conditioning therapies in STEMI between 2005 and 2013. Serial blood sampling was available in all studies with data regarding creatine kinase (CK), CK specific of cardiomyocytes (CK-MB) and cardiac troponin I. Our model allowed an accurate estimation of biomarker release as a surrogate marker of infarct size and a powerful assessment of conditioning treatments. This biomarker kinetic modelling approach identified CK-MB as the most accurate biomarker in determining infarct size and supports the development of limited sampling strategies that estimate total biomarker amount released with a lower number of samples. It will certainly be a useful add-on to future studies in the field of STEMI and cardioprotection.

## Introduction

In the context of myocardial ischemia-reperfusion injury, infarct size is a key predictor of subsequent major cardiovascular events^1, 2^. If it can be accurately determined using imaging techniques such as myocardial radionuclide scintigraphy or more recently cardiac magnetic resonance (CMR) imaging, actually considered to be the gold standard in infarct size assessment, the accessibility to these techniques is an important limitation in routine practice^3, 4^. Many centres worldwide including some participating in cardioprotection clinical trials in the context of acute myocardial infarction don’t have access to cardiac MRI technology. For the remaining centres, cardiac MRI is usually not available during off-business hours and non-working days. This renders the assessment of infarct size in the early phase of acute myocardial infarction still mainly dependent on serial blood measurements of biomarkers that are more likely to be available on a 24/7 basis. Necrosis markers commonly measured in routine practice are serum creatine kinase (CK), creatine kinase myocardial band specific of cardiomyocytes (CK-MB) and troponins I (cTnI) or T, the latter being considered as the gold standard currently used in the universal definition of myocardial infarction by both the European Society of Cardiology and the American College of Cardiology/American Heart Association^5, 6^.

According to updated state-of-the-art knowledge regarding cardiovascular diseases, the early impact of cardioprotective techniques/drugs on ischemia/reperfusion-induced lesions can be monitored through infarct size reduction^7, 8^. This is the reason why this parameter is a first-choice key endpoint in translational research on acute myocardial infarction. In the past decade, conditioning therapies (ischemic post-, remote and pharmacological with Cyclosporine A) were identified as major promising interventions to reduce infarct size and improve patients’ prognosis, though the latter remains to be demonstrated^9, 10^. Usually, the relevance of these conditioning techniques are assessed by measuring the total amount of necrosis marker released by the injured myocardium using the peak and/or the area under the concentration versus time curve (AUC) of CK, CK-MB and cTnI. These surrogate endpoints are used because of their availability and their known correlation with infarct size^11–13^. After patient’s inclusion, several blood samples are usually collected, and CK activity and cTnI concentrations measured from inclusion up to 72 hours. The AUC of CK and cTnI are calculated using trapezoidal rule, which is the reference method^14^. However, biomarker concentrations do not only include information on the amount of biomarker released by the lesion as it is influenced by its kinetics of release, distribution and elimination. These parameters present an important inter-individual variability. Therefore, AUC do not provide an accurate estimation of the amount of released necrosis marker. For instance, a patient with a lower than average elimination rate of a given biomarker will have an overestimated AUC compared to a median patient, thus blurring the results of cardioprotection studies.

One relevant way to estimate the total amount of marker released by the lesion into the bloodstream may be to use mathematical models describing its kinetics over time with input and output quantifications. These models, known as “compartmental models”, are commonly used to describe the pharmacokinetics (PK) of drugs.

Biomarker kinetic modelling was previously used to describe prostate-specific antigen decrease after adenomectomy^15﻿^, human chorionic gonadotrophin decrease in cancer patients treated with chemotherapy^16^, S100 calcium-binding protein (S100b) kinetics of decrease after traumatic brain injury^17^ and N-terminal pro B-type natriuretic peptide (NT-proBNP) kinetics to predict the risk of mortality following acute heart failure^18^. In pharmacokinetic modelling, the amount (dose) of the study drug administered to patients is known, whereas the amount of necrosis biomarker released by injured tissue is unknown and has to be estimated. The description of biomarker kinetics using modelling would allow the quantification of (i) the amount of biomarker released by injured tissue, (ii) the kinetics of this release, (iii) the distribution of biomarker in the body and (iv) the elimination of biomarker from serum. The quantification of all of these phenomena should allow an accurate quantification of the amount of biomarker released by injured tissue, a variable that could be of precious value in cardioprotection studies.

The main goal of the present study was to develop a kinetic model allowing the estimation of the total amount of necrosis biomarkers (CK, CK-MB, cTnI) released during myocardial ischemia-reperfusion injury from its concentrations measured in serum. We could derive from this information easy and accurate infarct size estimations that would serve in the evaluation of cardioprotective therapies.

## Results

### Patient population and data collection

Among the 246 patients included in clinical trials, 180 patients had a full biomarker measurement profile and were assessed, i.e. 43 (17 and 26), 57, 32, and 49 patients for “PC” studies, “PC CsA”, “PCNR” and “RIPOST-MI” studies, respectively (Table 1). Therefore the three full data sets, i.e. with CK, cTnI and CK-MB, included 132, 132 and 49 patients, respectively. Learning and validations subsets for CK and cTnI included 84 and 48 patients, respectively, whereas 32 and 17 patients were assigned for CK-MB, respectively.

**Table 1:** Summary of assessed patients characteristics.

Study parameters	CK and cTnI	CK-MB
Patients evaluable	132	49
Study arms	Control - 61	Control - 16
	Treatment - 71	RIPer - 17
		RIPer + IPOST - 16
Sex (female/male)	101/31	40/19
Diabetes (yes/no)	19/113	
Age (years)	58 [49–68]	59 [49–72]
Area at risk (% of ACS)	34.8 [27.0–46.5]	37.4 [30.5–45.7]
Body weight (kg)	75 [67–84]	76 [70–83]
Height (cm)	171 [165–176]	170 [166–175]
Glycemia (g/L)	1.31 [1.18–1.67]	1.40 [1.10–1.71]
Serum Creatinine (μmol/L)	83 [77–92]	78 [68–103]
Hemoglobin (g/dL)	14.2 [12.8–14.9]	14.9 [14.0–15.8]
AUC CK (IU.h/L)	74213 [44032–109236]	—
CK peak (IU/L)	2745 [1671–4418]	—
AUC cTnI (mg.h/L)	2607 [1452–4267]	—
cTnI peak (mg/L)	97 [46–161]	—
AUC CK-MB (IU.h/L)	—	5403 [3631–7742]
CK-MB peak (IU/L)	—	306 [196–451]

AUC, area under the concentration versus time curve; CK, creatine phosphokinase; cTnI, cardiac troponin I; CK-MB, creatine phosphokinase muscle-brain.

A total of 1965 CK, 1965 cTnI and 735 CK-MB measurements were available. Of notice, in the original publication from Staat *et al*., CK data only were reported although cTnI data had also been recorded and were included in this analysis.

### Biomarker kinetic analysis

Model building was made on learning subsets. Biomarker kinetic models allowed an estimation of total inputs of CK (CK_TOT_), cTnI (cTnI_TOT_) and CK-MB (CK-MB_TOT_) levels. The kinetics of these models were described and quantified separately from each other (Table 2). Elimination half-lives of CK, cTnI and CK-MB were computed using kinetic parameters estimates and were 11.2 h, 37.8 h and 7.2 h, respectively.

**Table 2:** Parameter estimates.

Biomarker		CK (IU/L)	cTnI (mg/L)	CK-MB (IU/L)
Parameter	Unit	Estimate (RSE %)	Estimate (RSE %)	Estimate (RSE %)
**B**_**TOT**_	**Biomarker**	**2120** (**23**)	**196** (**29**)	**167** (**18**)
Arm on B_TOT_	—	−0.43 (34)	−0.56 (38)	−0.75 (29)
AAR on B_TOT_	—	0.020 (30)	0.019 (39)	0.037 (24)
B_1_	biomarker	173 (12)	0.11 (41)	43.1 (36)
B_2_	biomarker	—	—	—
Fr	—	0.15 (31)	0.53 (14)	—
n_1_	nb of transit compart.	2.5 (7)	2.0 (10)	1.0 (19)
k_tr1_	h^−1^	0.68 (11)	0.29 (6)	0.34 (12)
n_2_	nb of transit compart.	50.7 (8)	4.2 (6)	—
k_tr2_	h^−1^	14.4 (7)	0.88 (1)	—
k_prod_	Biomarker/h	6.9 (11)	—	0.32 (8)
k_e_	h^−1^	0.062 (3)	0.097 (6)	0.096 (2)
k_12_	h^−1^	—	0.11 (8)	—
k_21_	h^−1^	—	0.044 (12)	—
ω_Btot_ (−)	—	0.65 (8)	0.80 (8)	0.51 (14)
ω_B1_ (−)	—	1.1 (8)	3.7 (18)	1.1 (29)
ω_B2_ (−)	—	—	—	—
ω_Fr_ (−)	—	0.79 (20)	0.59 (13)	—
ω_n1_ (−)	—	0.49 (11)	0.46 (11)	0.64 (23)
ω_Ktr1_ (−)	—	0.78 (10)	0.43 (14)	0.18 (22)
ω_n2_ (−)	—	0 (fixed)	0.25 (27)	—
ω_Ktr2_ (−)	—	0 (fixed)	—	—
ω_Kprod_ (−)	—	0.83 (10)	—	0.37 (15)
ω_Ke_ (−)	—	0.23 (10)	0.23 (15)	0.10 (14)
ω_12_ (−)	—	—	0.16 (30)	—
ω_21_ (−)	—	—	0.79 (14)	—
σ_add_ (unit of biomarker)	biomarker	9.3 (15)	0.82 (20)	0.19(20)
σ_prop_ (%)	%	0.099 (3)	0.15 (4)	0.12 (5)

CK, creatine phosphokinase; cTnI, cardiac troponin I; CK-MB, creatine phosphokinase muscle-brain; ω, interindividual standard deviation; σadd; additive error standard deviation; σprop, proportional error standard deviation; B_TOT_, total biomarker input released by lesion. AAR, area at risk; ACS, abnormally contracting segments.

All other parameters are explained in text.

#### Base models for CK, cTnI and CK-MB

The kinetic models that were tested included a release model with a single transit or a combination of two transit models, and a distribution/elimination model with one or two compartments. Therefore, four models were finally tested. Baseline biomarker production was removed from the model if it could not be estimated.

- CK data were best described using a two-transit mono-compartment model.
- cTnI data were best described using a two-transit two-compartment model, which is consistent with a previous study which reported a biphasic elimination of cTnI and two peaks^19^.
- CK-MB data were best described using a single transit mono-compartment model (Table 2).

**Figure 1:**
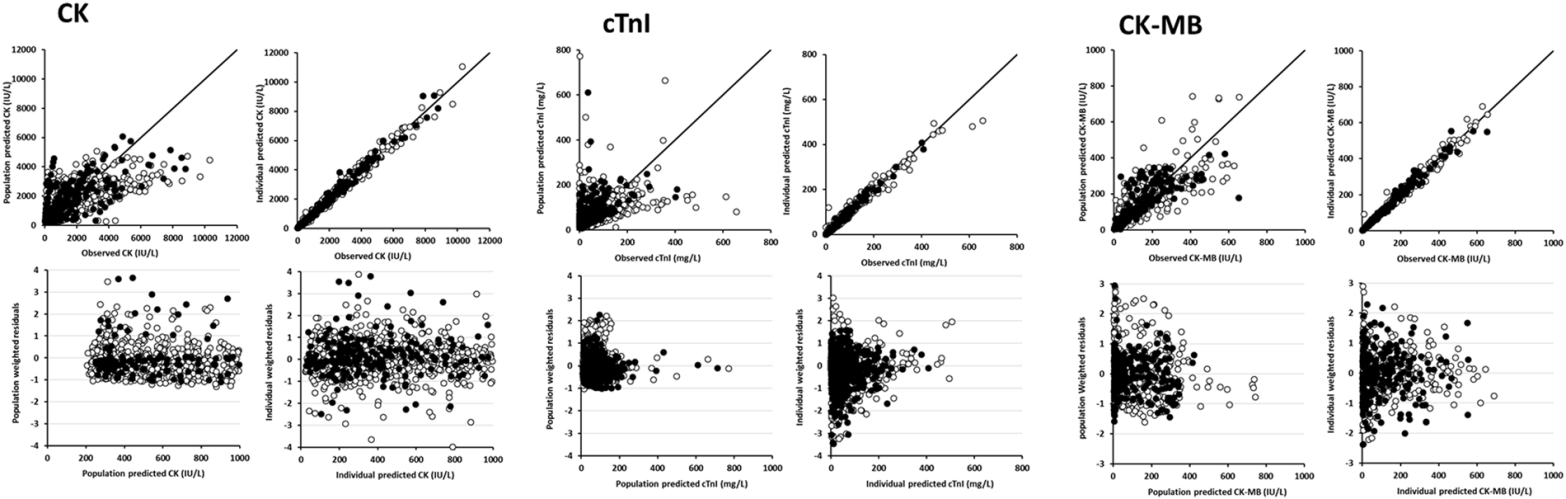
Diagnostic plots of creatine phosphokinase (CK, left), cardiac troponin I (cTnI, middle) and creatine phosphokinase muscle-brain (CK-MB) kinetic models. For each biomarker: observed *versus* model-predicted for population parameters (top-left) and for individual parameters (top-right). The line is the first bisector line. White and black circles are observed/predicted biomarker level couples for learning and validation subsets, respectively. population weighted residuals *versus* population predicted biomarker levels (bottom-left); individual weighted residuals *versus* individual predicted biomarker levels (bottom-right). White and black circles are predicted biomarker level couples and weighted residuals of learning and validation subsets, respectively. Typical parameters describe covariate-explained variability, whereas individual parameters describe both explained and unexplained variability. Population plots of CK and cTnI show a bias for population parameters, which disappears for individual parameters. The strong similarity of results obtained from learning and validation subsets demonstrate the good predictive performance of biomarker kinetic models.

**Figure 2:**
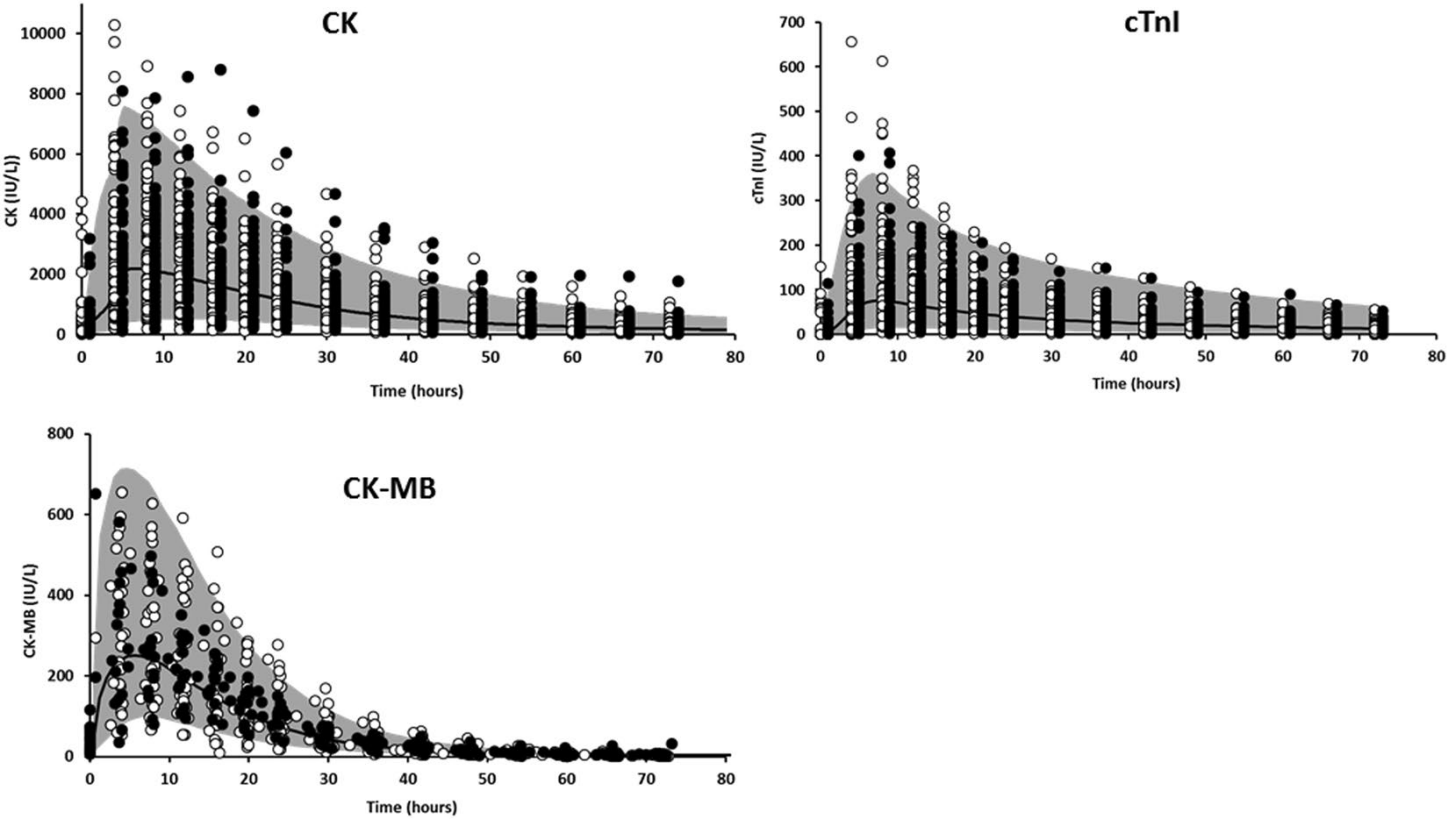
Prediction interval with 90% of kinetic profiles for creatine phosphokinase (CK), cardiac troponin I (cTnI) and creatine phosphokinase muscle-brain (CK-MB) kinetic models *versus* time. White and black circles are observed biomarker levels of learning and validation subsets, respectively.

The best error model was mixed additive-proportional for all biomarkers. Levels of CK, cTnI and CK-MB were adequately described by the kinetic models (Fig. 1), even if a bias was observed for CK and cTnI population parameters, which disappeared for individual parameters. Interindividual variances of some release model parameters could not be estimated and were therefore fixed to 0 (Table 2). All other parameters were estimated with good precision (with relative standard errors <30%, Table 2). Plots of residuals (Fig. 1) and of prediction intervals (Fig. 2) showed no obvious bias or model misspecification for any biomarker.

#### Covariate models

Regarding the association between body weight, age, AAR, study arm, and the total input of biomarker released by the lesion CK_TOT_, cTnI_TOT_ and CK-MB_TOT_, the univariate step showed that (α < 0.1):

(i) CK_TOT_ increased with AAR (LRT = 10.2, p = 0.0014) and was lower in the conditioning treatment arms (LRT = 4.5, p = 0.034).
(ii) cTnI_TOT_ increased with AAR (LRT = 12.1, p = 0.0061) and was lower in conditioning treatment arms (LRT = 3.4, p = 0.064).
(iii) CK-MB_TOT_ increased with AAR (LRT = 8.7, p = 0.0032) and was lower in RIPer and/or IPOST treatment arms (LRT = 6.7, p = 0.0098).

**Figure 3:**
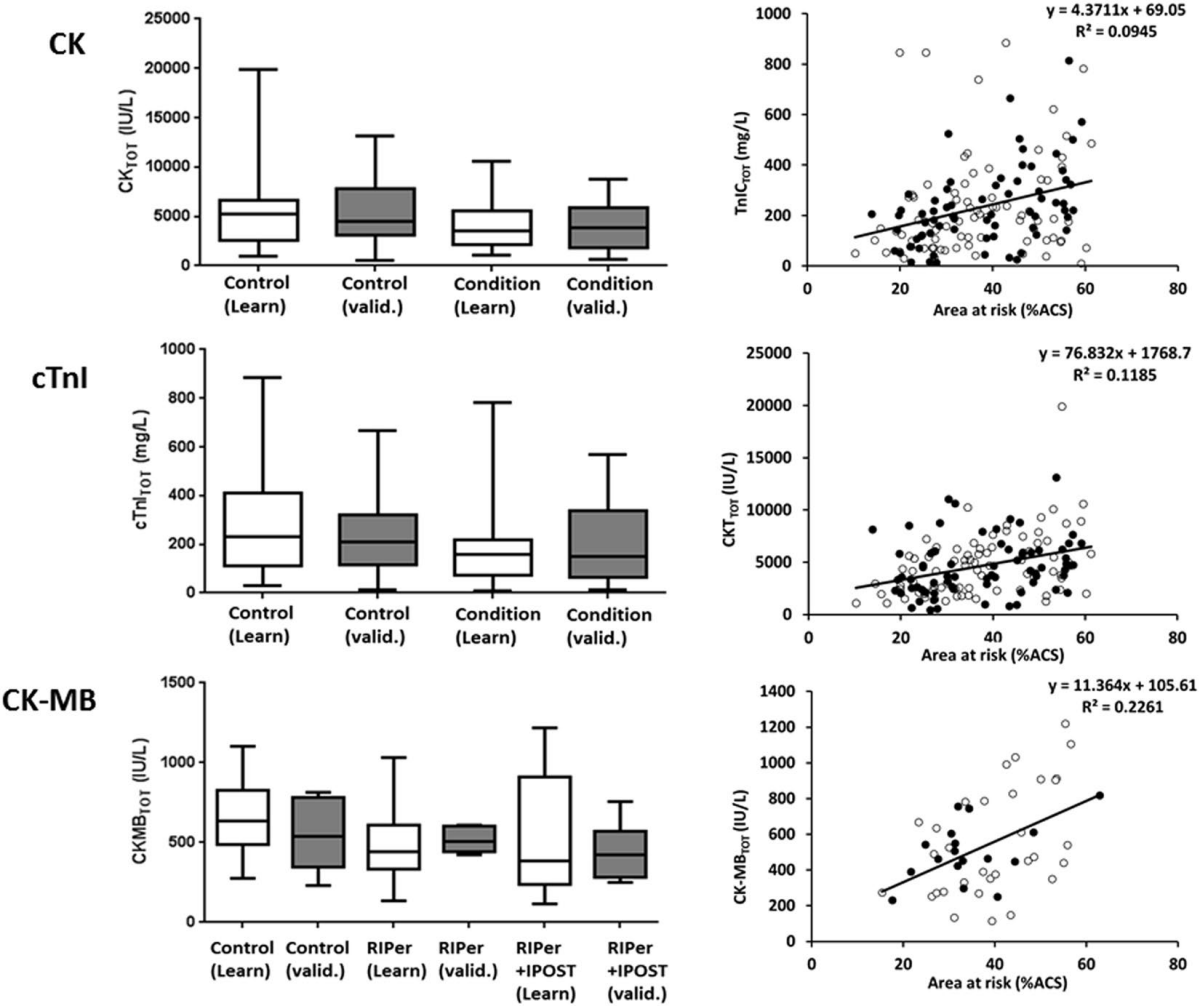
Individual parameter estimates for creatine phosphokinase (CK, top), cardiac troponin I (cTnI, middle) and creatine phosphokinase muscle-brain (CK-MB, bottom) kinetic model parameters *versus* conditioned arm (left) and area at risk (AAR, right). For control arm, whisker boxes display, from bottom to top, 5^th^, 25^th^, 50^th^, 75^th^ and 95^th^ individual parameter value percentiles. White and grey boxes are learning and validation subset results, respectively. For AAR, circles are covariate/individual parameter couples and line is regression line with equation and coefficient of determination (R^2^). White and black circles are AAR/biomarker levels of learning and validation subsets, respectively.

The multivariate step showed that (α < 0.01, Table 2, Fig. 3):

(i) CK_TOT_ increased with AAR (LRT = 11.06, p = 0.0009), and was lower in the conditioning treatment arms (LRT = 8.4, p = 0.0037).
(ii) cTnI_TOT_ increased with AAR (LRT = 9.0, p = 0.0027) and was lower in the conditioning treatment arms (LRT = 5.8, p = 0.016).
(iii) CK-MB_TOT_ increased with AAR (LRT = 16.9, p = 3.8.10^−5^) and was lower in RIPer and/or IPOST treatment arms (LRT = 12.0, p = 0.00054). Because the RIPer arm was not significantly different from the RIPer + IPOST arm (p = 0.89), these arms were merged (Table 2): the reference category was control and RIPer arms and the other category was RIPer/RIPer + IPOST.

#### External validation

External validation was made on validation subsets. Individual kinetic model estimates, AUC values and diagnostic plots showed no difference between learning and validation subset results (Figs 1, 2 and 3). This shows that both model structures and estimated parameter distributions allow satisfactory predictions of biomarker kinetic profiles for patients independent from learning data set.

#### Estimation of area under the biomarker concentration versus time curve (AUC)

**Figure 4:**
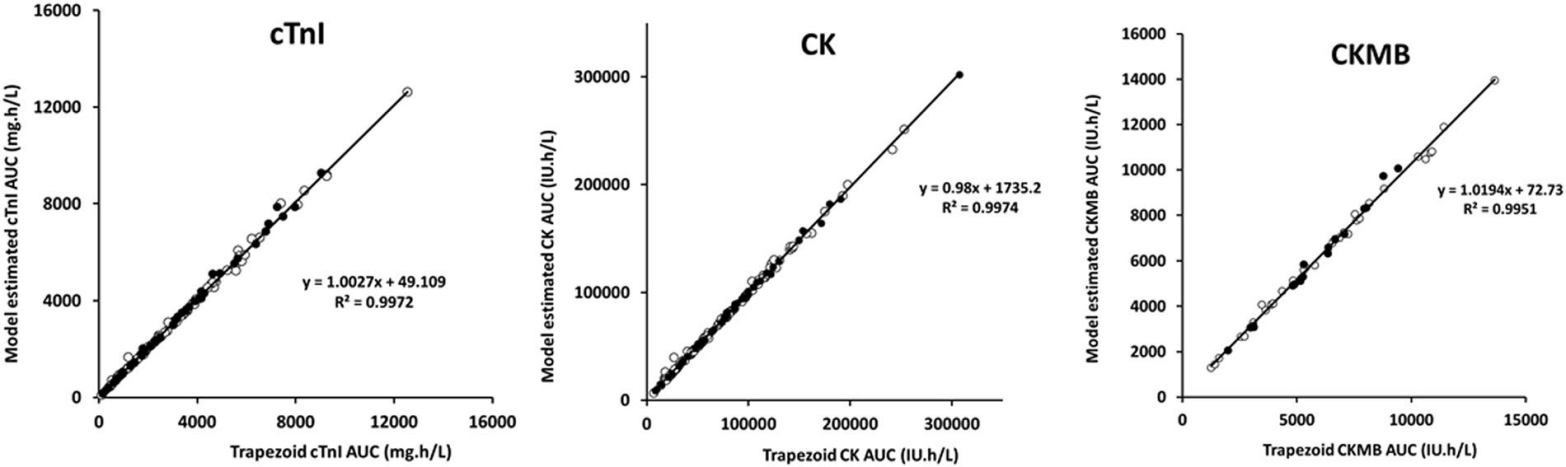
Linear correlations between model-predicted area under the biomarker curve (AUC) and trapezoidal rule AUC for creatine phosphokinase (CK, left), cardiac troponin I (cTnI, middle) and creatine phosphokinase muscle-brain (CK-MB). All correlation coefficients are above 99%. White and black circles are trapezoid/model-estimated AUC of learning and validation subsets, respectively.

For CK, cTnI and CK-MB, the estimations of AUC using trapezoidal rule and computed using individual model parameter estimates were highly correlated (R^2^ ≥ 99%, Fig. 4) with no bias, showing that model-based computation provided similar AUC estimations.

#### Association of covariates with trapezoidal rule AUC

Univariate general linear models showed that trapezoidal rule AUC of CK and CK-MB (Table 3) were significantly associated with the conditioning therapies (p = 0.021 and 0.020, respectively), while AUC of cTnI was not (p = 0.21).

**Table 3:** P-values of association between study arm and biomarker amount.

Biomarker	CK		cTnI		CK-MB	
Technique	GLM	NLMM	GLM	NLMM	GLM	NLMM
Univariate	0.021	0.024	0.21	0.064	0.02	0.0098
Multivariate	0.011	0.00039	0.24	0.0087	0.0064	0.000045

CK, creatine kinase; cTnI, cardiac troponin I; CK-MB, creatine phosphokinase muscle-brain; GLM, association between biomarker AUC and study arm using general linear model; NLMM, association between study arm and estimated biomarker amount using nonlinear mixed modelling.

In learning subsets, multivariate general linear models showed that (Table 3):

- trapezoidal rule AUC of CK was significantly associated with the conditioning treatment arms and AAR (p = 0.033 and 0.00011, respectively).
- trapezoidal rule AUC of cTnI was significantly associated with AAR (p = 0.00065), but not with conditioning treatment arms (0.27).
- trapezoidal rule AUC of CK-MB was significantly associated with the conditioning treatment arms and AAR (p = 0.015 and 0.000193.

Kinetic biomarker modelling had a higher power for quantification of the conditioning treatment effect on biomarker release than trapezoidal rule AUC (Table 3). Of note, biomarker kinetic modelling approach showed a significant influence of conditioning treatment arm on cTnI release, which was not significant using general linear modelling.

## Discussion

This study is the first to describe a simple and reliable estimation of the amounts (B_TOT_) of necrosis biomarkers (CK, cTnI, CK-MB) that are released by injured myocardium, obtained using a mathematical description of their kinetics.

Translational research in the field of cardioprotection has been quite often unsuccessful in the past two decades probably due to the choice of surrogate endpoints that were not relevant or difficult to measure accurately. Major adverse cardiac and cerebrovascular events (MACCE) are considered the most robust endpoints. However, evaluating the effect of a treatment on MACCE reduction after myocardial infarction in patients receiving up-to-date treatments can only be performed in large clinical trials enrolling a large number of patients. Proof-of-concept studies often use softer endpoints such as surrogate endpoints. In acute myocardial infarction studies the usual surrogate endpoint is infarct size reduction that can be estimated using imaging techniques and/or necrosis biomarkers measurements but with a lack of accuracy. Our kinetic modelling provides the missing accuracy to biomarkers assessment and this improved infarct size estimation allows a finer evaluation of the impact of conditioning therapies than that reported in the original papers. We demonstrate that the association between conditioning therapies and B_TOT_ is stronger than the association between conditioning therapies arm and trapezoidal rule AUC (Table 3). In addition, our model was able to detect an association between conditioning therapies and cTnI release (p = 0.0087) whereas this association was not detected using trapezoidal rule AUC in the original published studies (p = 0.064).

The strong similarity of results obtained in learning and validation subsets shows that our models are able not only to describe biomarker (i.e. CK, cTnI and CK-MB) kinetic data (learning subset), but also to predict such kinetics of data, which were not involved in model building and parameter estimation. Our models should therefore be reproducible in other studies and suitable to estimate individual kinetic parameters and AUC of biomarkers of new patients and/or new studies without model building or parameter distribution estimation steps.

Kinetic modelling provided lower p-values for association of biomarker with conditioning therapies, AAR and age, showing higher statistical power compared to general linear modelling (ANCOVA). This approach should provide accurate infarct size assessment in STEMI clinical trials and may open the way to trials with reduced numbers of subjects in a close future. In addition, these data suggest that our mathematical model is probably the best way to determine infarct size in STEMI patients in daily practice, biomarkers assessment being available in every single centre worldwide managing such patients. Indeed, because the amounts of released biomarkers are correlated to infact size (Fig. 3), and provide more powerful tests than comparison of AUC, these amounts are likely to be correlated to infarct size measured by MRI. However, future studies would be needed to quantify the actual correlation between biomarker amounts and MRI measurements.

An interesting point is the comparison that can be made between the different necrosis biomarkers used to estimate infarct size in these studies. For CK and even more for cTnI, measurement of AAR may still be necessary whatever the model used (multivariate biomarker kinetic or general linear models); however, the use of biomarker kinetic modelling appeared to provide powerful testing of association between conditioning therapies and CK-MB release (p = 0.0098) without the use of AAR.

The kinetics of CK-MB is described using a simpler model than CK or cTnI, and the association between estimated total CK-MB released amount (CK-MB_TOT_) and AAR is stronger (R^2^ = 0.23, p = 3.8.10^−5^) than that observed between CK_TOT_ or cTnI_TOT_ and AAR (R^2^ = 0.095, p = 0.0009 and 0.12, p = 0.00027, respectively, Fig. 4). This is consistent with a stronger association of conditioning therapies with CK-MB release compared to CK or cTnI release, despite the lower number of subjects for CK-MB analysis, meaning that CK-MB are more accurate than CK or cTnI for the assessment of the cardioprotective effect of conditioning therapies.

The strength of CK-MB release in assessing the effects of conditioning therapies without the need to measure AAR may be a breakthrough in this research field as accurate assessment of AAR is almost impossible to obtain in daily clinical practice at the acute phase of myocardial infarction. The AAR estimation performed in several publications studying ischemic postconditioning remains questionable as it rests on data from a left biplane ventriculography that (i) is not routinely performed during PCI, (ii) is subjected to errors and bias while visually estimating the abnormal contracting circumference, and (iii) increases the risk of contrast-induced nephropathy in these already high-risk STEMI patients whose renal function is often unknown at the time of management^20, 21^. Moreover, recent data regarding MRI and the assessment of AAR suggest that it may overestimate AAR when performed after reperfusion occurred, as myocardial oedema significantly increases at the time of reperfusion. This particular point could render more complex the interpretation of ischemic postconditioning efficacy as it was shown to reduce myocardial oedema compared to the control group^22^. A recent paper from Engblom *et al*. emphasized the potential of cardiac MRI in assessing both infarct size and AAR at the early phase of STEMI and suggested that systematic use of this technology in cardioprotection studies would result in a reduction of the number of patients needed, yet again access to this technique is still sparse^23^. Such an attitude would confine the realization of subsequent trials to a limited number of centres, yet increasing the risk of observing a non-generalizable population effect. In addition, they showed that the time of realization of cardiac MRI from the onset of myocardial infarction impacts on the determination of the AAR, increasing the risk of bias and finally the number of needed patients as compared to an ideal 100% standardized situation where all patients would be tested on the very same day. Thus, the observed accuracy and relevance of kinetics of CK-MB in assessing infarct size without the need to measure AAR would regenerate the use of this biomarker. CK-MB were nearly abandoned in the past decade in favour of cardiac troponin, a change in practice determined by the demonstrated superiority of cardiac troponin over CK-MB in acute coronary syndrome risk stratification especially in patients experiencing minor degrees of ischemia^24, 25^. This demonstrated superiority led in 2000 to a new universal definition of myocardial infarction with a significant benefit on 10 years mortality rate^26^. Nonetheless, if troponin measurement is the best marker for the diagnosis of acute coronary syndrome, according to our work, it appears less correlated to infarct size than CK-MB. This is of major importance since infarct size is considered the best predictor of mortality after acute myocardial infarction. Our data are in keeping with real-life data published by Chin *et al*. who suggested that peak CK-MB had a higher discriminative value than peak cTnI in predicting cardiac mortality^27^. On the contrary, Chia *et al*. reported that cTnI assessed at 72 hours following primary PCI in the context of STEMI due to LAD occlusion was the best biomarker to estimate infarct size, contractile function and thus predict future cardiovascular events, but their series was considerably smaller than Chin’s^28^. We do not believe that the spread of hypersensitive troponins, mainly useful in an earlier identification of non-STEMI patients, adds anything to the matter, yet we could not evaluate them in our model as they were not available at the time of realization of the trials considered here. Recent publications suggest that copeptin may be a better marker of myocardial ischemia than cardiac troponin, being more sensitive and earlier detectable. This is however an indirect marker of myocardial ischemia, also increased in other situations of acute diseases, i.e. less specific than troponin, though troponin release is not specific of myocardial necrosis. Of note, recent data suggest that copeptin concentration also correlates with infarct size in STEMI patients^29^.

Last but not least, the powerful testing of association of conditioning therapies and biomarker release through biomarker kinetic modelling is based on population compartmental analysis, an approach extensively used in pharmacokinetic analysis. Our modelling strategy provides not only a complex yet necessary description of biomarker release with time, but also the quantification of the interindividual variability of biomarker kinetics. This approach requires specific technical expertise to be implemented, but could be the basis of the development of limited sampling strategies, where both total biomarker released amount and AUC would be determined using a lower number of blood samples. This point is also of major clinical importance as it has been demonstrated that subjects participating in clinical trials with repetitive blood sampling are more prone to iatrogenic anaemia^30^, and anaemia was reported to be associated with a negative prognosis in coronary heart disease patients^31, 32^.

In conclusion, we demonstrated for the first time that the use of a mathematical model to describe necrosis marker kinetics allowed simple and accurate infarct size estimations, opening the way for future cardioprotection studies with increased power allowing reduced number of patients and reduced blood sampling. While cardiac troponins were praised in the last decades as the gold standard for myocardial ischemia detection, CK-MB were in our work the biomarkers with the strongest association with infarct size and consequently the best prognosis value in STEMI patients in the global context of reduced cardiac MRI availability. Further, CK-MB was best correlated with the AAR, thereby attenuating the concern related to the current absence of measurement of this major determinant of infarct size in infarct size reduction trials. This model is likely to be a useful add-on to future studies in the field of cardioprotection.

## Methods

### Patient population and data collection

#### Clinical trials

Data were prospectively collected in ST-segment elevation myocardial infarction (STEMI) patients involved in four clinical trials for which local ethics committees had approved the protocols (ethic committee of the Hospices Civils de Lyon for PC, PC-CsA and PCNR and ethic committee of Angers University Hospital for RIPOST-MI) in which all patients had given written informed consent. All studies were performed in accordance with the Declaration of Helsinki (revised version, 1996), the European Guidelines for Good Clinical Practice (version 11, July 1990), and French laws.

PC (ischemic postconditioning) studies^33, 34^. The first study was a prospective, randomized, multicenter, open-label, controlled study. It was a proof-of-concept clinical trial aiming at demonstrating the safety and efficacy of ischemic postconditioning at the time of reperfusion by primary percutaneous coronary intervention (PCI) in STEMI patients presenting within 6 hours a STEMI symptoms onset and with an occluded (TIMI 0 flow grade) left anterior descending (LAD) coronary artery or right coronary artery without evidence of coronary collaterals. The area at risk (AAR) was estimated using a biplane left ventricular angiography in order to measure the circumferential extent of abnormally contracting segments (ACS)^35–38^. Thirty-three patients were included in the study: 14 in the control group, 16 in the postconditioned group and 3 patients who were excluded according to the prespecified exclusion criteria. Aside from the ST-segment resolution following PCI and blush grade (as a sign of a quality reperfusion), the authors performed serial serum CK measurements with AUC calculations.

The second study evaluating the impact of ischemic postconditioning from the Ovize group was also prospective, randomized, multicenter, open-label, controlled, and used the same experimental protocol with an extended follow-up consisting in a ^201^thallium single photon emission computed tomography at 6 months and a transthoracic echocardiography at 1 year. 38 patients were included, 21 were assigned to the control group and 17 to the ischemic postconditioning group.

PC-CsA study^39^. This clinical trial from the Ovize group was a prospective, multicenter, randomized, single-blinded, controlled study aiming at determining the effect of the mitochondrial permeability transition pore inhibitor Cyclosporine A (CsA) on infarct size in a population of STEMI patients similar to that of the above-described PC studies. The primary endpoint was infarct size measured by AUC of CK and cTnI, the secondary endpoint was infarct size assessed by CMR imaging performed at day 5 following acute myocardial infarction. Data were reported for 58 patients, 30 randomized in the control group and 28 in the CsA group.

PCNR study^22, 40^. This prospective, multicenter, randomized open-label, controlled study aimed at evaluating the effect of ischemic postconditioning on no-reflow, myocardial oedema and infarct size in a population of STEMI patients, similar to the patients included in the above-reported PC studies. The main difference between populations was the myocardial infarction topography: as opposed to PC studies that considered only STEMI resulting from LAD or right coronary occlusion, this PCNR study also considered patients presenting an occlusion of the left circumflex coronary artery in case of left circulation dominance. In addition to serial blood sampling aiming at determining infarct size using the peak and AUC of serum CK, all patients underwent CMR imaging 48–72 hours after admission for assessment of infarct size, myocardial edema and early and late microvascular obstruction. Sixty-two patients were randomized but the authors reported the CMR results for 50 patients only as 10 patients refused to undergo CMR imaging and CMR study was uninterpretable in 2 other patients.

RIPOST-MI study^41^. This was a prospective, multicenter, randomized, open-label, and blinded-endpoint study. It aimed at (i) determining if a remote ischemic perconditioning (RIPer) procedure, when initiated on admission in the catheterization laboratory, would reduce infarct size in STEMI patients treated with primary PCI within the first 6 h of symptoms onset and (ii) investigating the effect of RIPer and local ischemic postconditioning (IPost) combination in terms of infarct size reduction. A total of 55 patients were eligible for CK-MB AUC analysis: 17 patients were allocated to the control group, 18 to the RIPer group and 20 to RIPer + Ipost. In addition to serial blood CK-MB measurements, the AAR was determined by a left biplane ventriculography in order to assess the circumferential extent of ACS as previously described.

In all studies, blood samples were collected prior to PCI, every 4 h in the first 24 h following PCI, and every 6 h over the following 48 h. CK and cTnI data were obtained from PC, PC-CsA and PCNR trials and were pooled for the kinetic modelling. CK-MB levels were obtained from the RIPOST-MI study only.

#### Data splitting

Data from “RIPOST-MI” trial studies, which were made of CK-MB measurements, were assessed separately. Three full data sets, i.e. with CK, cTnI and CK-MB variables, were built. With the aim of evaluating the predictive performances of kinetic models, data sets were randomly assigned into learning (2/3 patients) and validation (1/3 patients) subsets. Learning and validation subsets were used for internal and external validation kinetic models, respectively.

### Biomarker kinetic analysis

#### Kinetic models

The objective of kinetic analysis was to estimate the input of biomarker released by the lesion. Kinetics of CK, CK-MB and cTnI was described using models derived from commonly used pharmacokinetic compartmental models^42^. The kinetics of these biomarkers was developed and its parameters estimated separately, i.e. separate models were used for each biomarker. These models describe absorption, distribution, metabolism and excretion (“ADME”) steps. Using these models, drugs are described as being distributed in a central compartment, corresponding to bloodstream and loci rapidly equilibrating with it, and one or more peripheral compartments, corresponding to other loci, which are not immediately in equilibrium with central compartment.

*Biomarker release*. Similarly to drug absorption (i.e. the passage from absorption site to bloodstream), the release of biomarkers by injured tissue (i.e. the passage of biomarker from tissue to bloodstream) is delayed in time. This non-instantaneous release may be described using models that are sometimes used to describe drug absorption. Among these models, transit absorption models^43^ have been used to describe a delay in absorption occurring as drugs travel through a certain number (non-integer) of “transit compartments”, as it is the case for CsA^44^, mycophenolate^45^ or rifampicin^46^. This transit model was already used to describe the kinetics of S100 calcium-binding protein, a biomarker used in traumatic brain injury^17^. For each patient, the origin of time is set to the first blood sample (t_0_) and biomarker release function f(t) is written as follows:

1 $$f(t)=\frac{{({k}_{tr}.t)}^{n}.{e}^{-{k}_{tr}.t}}{{\rm{\Gamma }}(n)}$$

where n is the number (not necessarily integer) of transit compartments, k_tr_ is the transit rate constant, t is the time from the first blood sample and Γ(n) is gamma function. The release model R(t) may be:

- either a single transit model: R(t) = B. f(t)
- or a combination of two transit models (similarly to the absorption of mycophenolate) as follows:

2 $$R(t)=[F.{f}_{1}(t)+(1-F).{F}_{2}(t)]$$

where B is the input of biomarker released for t > t_0_, F is the proportion of biomarker amount released as described by f_1_(t), the first release model, and f_2_(t), the second release model. Both f_1_(t) and f_2_(t) are release functions with n_1_ and n_2_ transit compartments, respectively, and k_tr1_ and k_tr2_ are their respective transit rate constants. The parameters n and k_tr_ (for the single release function), or n_1_, n_2_, k_tr1_, k_tr2_ and F (for the combination of two release functions) are structural parameters that had to be estimated.

The main parameter, which is the total input of biomarker released, was the sum of the biomarker already released before t_0_ (B_0_), and of the one released after t_0_ (B). Under the assumption of homogenous distribution of biomarkers in bloodstream, this parameter is proportional to the total amount of biomarker (B_TOT_), i.e. B_TOT_ = B_0_ + B.

*Biomarker distribution and elimination*. One and two compartment models with first-order distribution and elimination rate constants were tested (Fig. 1).

The mono-compartment model is written as follows:

3 $$\frac{dB}{dt}=R(t)+{k}_{prod}-{k}_{e}.B$$

where B is the biomarker input after t_0_, f(t) is the release function, k_e_ is the elimination rate constant and k_prod_ zero-order (constant infusion) is the baseline biomarker production (i.e. independent from disease). Initial value of B(0) could not be given the value of zero because biomarker release started before the first blood sample and was B(0) = B_0_. In this model, k_prod_, k_e_ and B_0_ are structural parameters that had to be estimated.

The two-compartment model is written as follows:

4 $$\frac{d{B}_{1}}{dt}=R(t)+{k}_{prod}-({k}_{e}+{k}_{12}).{B}_{1}+{k}_{21}.{B}_{2}$$

5 $$\frac{d{B}_{2}}{dt}={k}_{12}.{B}_{1}+{k}_{21}.{B}_{2}$$

where B_1_ and B_2_ are biomarker levels in central and peripheral compartments respectively, f(t) is the release function, k_12_ and k_21_ are the central to peripheral, and peripheral to central distribution rate constants. Initial values B_1_(0) and B_2_(0) (non-zero) had to be estimated as typical parameters. The amount of biomarker released before t_0_ is B_0_ = B_1_(0) + B_2_(0). Similarly to the mono-compartment model, k_12_, k_21_, B_1_(0) and B_2_(0) are structural parameters that had to be estimated.

#### Model development

The development of kinetic models was made on learning subset.

*Population approach*. Population modelling has been used to describe pharmacokinetic data since the early 70’s. If individual modelling is used to estimate the parameters of interest (*e*.*g*. the drug clearance in pharmacokinetics) at the “individual level”, *i*.*e*. for each individual taken separately from the others, the basic principle of population modelling is to estimate parameters at the “population level”^47^. The main goals of population modelling are to determine the distribution of the values of the parameters of interest in a population and to quantify the influence of the individual sources of variability in this population. Using a population approach, data from all individuals in a given population are computed simultaneously to estimate the interindividual distribution of parameters of interest. This interindividual distribution allows the quantification of (i) the “mean” (referred as “typical”) value of each parameter, (ii) the interindividual variability (referred as “interindividual variance”) and (iii) the influence of individual factors on interindividual variability (referred as “covariates”).

*Software*. Biomarker kinetic data were analysed by a population approach using the nonlinear mixed-effects program MONOLIX 4.3.2 software (Lixoft®, Saclay, France), which combines the stochastic expectation-maximization (SAEM) algorithm and a Markov Chain Monte-Carlo procedure for likelihood maximization. To ensure the best possible convergence, a large number of iterations (1000 for K1 and 250 for K2) was used. K1 and K2 refer to the SAEM procedure of Monolix, called “iterative kernels”. During K1, the sequence of step sizes is constant, which allows the exploration of the parameter space. During K2, the step sizes decrease to ensure convergence. Five Markov chains were used, and simulated annealing was used to improve the convergence of the SAEM algorithm towards the global maximum of the likelihood. Each run was performed three times to ensure that estimated parameters and likelihood remained stable. The random seed was changed between each of the three runs.

*Structural model design*. Biomarker concentrations were described with compartmental models with transit gamma release. One or two compartment models with first-order distribution constants were first tested. Then models with 2, 3, 4 and 5 transit compartments were tested. Structural models were compared using Akaike’s information criterion (AIC), defined as: AIC = OFV + 2.p, where OFV is the value of the objective function and p is the number of model parameters to estimate. The use of AIC is based on the parsimony principle aiming at a satisfactory fitting of the data with a small number of parameters. The OFV was –2.ln-likelihood (−2 LL). The model with the lowest AIC was selected.

*Interindividual model*. The interindividual variability of pharmacokinetic parameters was described using an exponential model: θ_i_ = θ_TV_. exp(η_i_), where θ_i_ is the estimated individual parameter, θ_TV_ is the typical value of the parameter and η_i_ is the random effect for the i^th^ patient. The values of η_i_ were assumed to be normally distributed with mean 0 and variance ω^2^.

*Error model*. Additive, proportional and mixed additive-proportional models were tested. For example, the combined additive-proportional model was implemented as follows: Y_O,ij_ = Y_P,ij_.(1 + ε_prop,i_j) + ε_add,ij_ where Y_O,ij_ and Y_P,ij_ are observed and predicted j^th^ marker measurements for the i^th^ patient, respectively, and ε_prop,ij_ and ε_add,ij_ are proportional and additive errors, which are assumed to follow a Gaussian distribution with mean 0 and variances σ_prop_^2^ and σ_add_^2^, respectively.

*Covariates*. The influence of three individual potential factors of variability on the distribution of B_TOT_ was tested:

- continuous covariate: AAR (% ACS)
- discrete covariates: conditioning therapies.
  - PC, PC_CsA and PCNR trials: study arm was coded as 0 (reference, control arm) or 1 (conditioned arm, i.e. ischemic postconditioning for PC and PCNR and pharmacological postconditioning with CsA for PC_CsA)
  - RIPOST-MI trial: study arm was coded as 0 (reference, control arm), 1 (remote ischemic preconditioning, RIPer) or 2 (RIPer + local ischemic postconditioning, IPost).
  The influence of a discrete covariate (ARM) on θ_TV_ was implemented as follows:
- For the reference category: ARM = 0, ln(θ_TV_) = ln(θ_ARM_ = 0).
- For another category: ln(θ_TV_) = ln(θ_ARM_ = 0) + β_ARM_ = x, where θ_ARM_ = 0 is the value of θ for the control arm, and β_ARM_ = x is the x^th^ value of θ_TV_ for the other category.

Area at risk was tested as follows: θ_TV_ = θ_0_. exp(β_AAR_.AAR), where β_AAR_ quantifies the influence of AAR on θ.

*Model comparison and covariate selection*. Interindividual, residual and covariate models are chosen by comparing 2 nested models, one with parameters, the difference of OFV is calculated. The difference in OFV is tested using a likelihood ratio test (RLT), i.e. the difference of OFV is compared to a chi-square law. However, LRT tests can be used only if the 2 models are nested, i.e. one model is a particular case of the other one. The influence of a covariate is tested by comparing OFVs of a model including the covariate (M1) and of a model without the covariate (M0). The value of OFV_M1_ − OFV_M0_) is assumed to follow a χ^2^ distribution and is therefore compared to a chi-square law with one degree of freedom for a given alpha risk.

The influence of patient characteristics (covariates) was assessed in two steps:

*Univariate step*. The influence of each factor on pharmacokinetic parameters associated with interindividual variability was tested. Covariates were separately included into the base model. Covariates showing a significant influence (α < 0.1) were included in the model (full model).

*Multivariate step*. A backward stepwise elimination was performed: the covariates of the full model were removed one by one. Covariates whose removal resulted in a statistically significant increase in the OFV (α < 0.01) were retained in the model. In RIPOST-MI trial, three arms were available and three values were therefore available for each covariate. If two categories were not significantly different, they were merged.

#### Model estimation of area under the biomarker concentration versus time curve (AUC)

To confirm that our pharmacokinetic model provides AUC estimations similar to those calculated by trapezoidal rule, individual AUCs for all biomarkers were (i) calculated using trapezoidal rule and (ii) computed using individual model parameter estimates. Trapezoidal rule AUC and model-computed AUC were compared using the coefficient of determination (R^2^).

#### Model validation

*Internal validation*. The goodness-of-fit was assessed for each model by plotting population-predicted (PRED) and individually predicted (IPRED) concentrations versus observed concentrations (DV) and IPRED and DV versus time. Population predictions were obtained using typical parameters, which include explained variability (i.e. population estimates and covariates), whereas individually predicted concentrations were obtained using individual parameters, which include both explained and unexplained (i.e. the random effects ηi for each PK parameter). In addition, the goodness-of-fit was evaluated by the distribution of residuals evaluated by graphical inspection of population (PWRES) and individual (IWRES) weighted residual distributions, visual predictive checks (VPC). These residuals should follow a standard normal distribution (i) to confirm a satisfactory description of the data using the model and (ii) to allow LRT tests.

*External validation*. This step was made to assess the predictive performances of the kinetic models. The validation subset was therefore not used for parameter estimation. The interindividual distribution of parameters determined during the model development step was used to estimate individual parameter values (kinetic parameters and AUC) for each patient.

Evaluation of model on validation subset was made by the goodness-of-fit procedures described above.

#### Association of covariates with trapezoidal rule AUC

The association of covariates with trapezoidal rule AUC of CK, cTnI and CK-MB was assessed using a general linear model (“analysis of covariance”, ANCOVA) in learning subsets. Dependent variable was trapezoidal rule AUC, and independent variables were age, AAR and conditioning therapy. The association of these factors with trapezoidal rule AUC was tested using Fisher F-tests. The analysis was made using R 3.2.2 (Vienna, Austria). The general linear model and biomarker kinetic modelling results were compared.
